# Sirtuin 3, a New Target of PGC-1α, Plays an Important Role in the Suppression of ROS and Mitochondrial Biogenesis

**DOI:** 10.1371/journal.pone.0011707

**Published:** 2010-07-22

**Authors:** Xingxing Kong, Rui Wang, Yuan Xue, Xiaojun Liu, Huabing Zhang, Yong Chen, Fude Fang, Yongsheng Chang

**Affiliations:** 1 The National Laboratory of Medical Molecular Biology, Institute of Basic Medical Sciences, Chinese Academy of Medical Sciences and Peking Union Medical College, Beijing, China; 2 Hubei Province Key Laboratory of Biotechnology of Chinese Traditional Medicine, Hubei University, Wuhan, China; Virginia Commonwealth University, United States of America

## Abstract

**Background:**

Sirtuin 3 (SIRT3) is one of the seven mammalian sirtuins, which are homologs of the yeast *Sir2* gene. SIRT3 is the only sirtuin with a reported association with the human life span. Peroxisome proliferator-activated receptor γ coactivator-1α (PGC-1α) plays important roles in adaptive thermogenesis, gluconeogenesis, mitochondrial biogenesis and respiration. PGC-1α induces several key reactive oxygen species (ROS)-detoxifying enzymes, but the molecular mechanism underlying this is not well understood.

**Results:**

Here we show that PGC-1α strongly stimulated mouse Sirt3 gene expression in muscle cells and hepatocytes. Knockdown of PGC-1α led to decreased Sirt3 gene expression. PGC-1α activated the mouse SIRT3 promoter, which was mediated by an estrogen-related receptor (ERR) binding element (ERRE) (−407/−399) mapped to the promoter region. Chromatin immunoprecipitation and electrophoretic mobility shift assays confirmed that ERRα bound to the identified ERRE and PGC-1α co-localized with ERRα in the mSirt3 promoter. Knockdown of ERRα reduced the induction of Sirt3 by PGC-1α in C_2_C_12_ myotubes. Furthermore, Sirt3 was essential for PGC-1α-dependent induction of ROS-detoxifying enzymes and several components of the respiratory chain, including glutathione peroxidase-1, superoxide dismutase 2, ATP synthase 5c, and cytochrome *c*. Overexpression of SIRT3 or PGC-1α in C_2_C_12_ myotubes decreased basal ROS level. In contrast, knockdown of mSIRT3 increased basal ROS level and blocked the inhibitory effect of PGC-1α on cellular ROS production. Finally, SIRT3 stimulated mitochondrial biogenesis, and SIRT3 knockdown decreased the stimulatory effect of PGC-1α on mitochondrial biogenesis in C_2_C_12_ myotubes.

**Conclusion:**

Our results indicate that Sirt3 functions as a downstream target gene of PGC-1α and mediates the PGC-1α effects on cellular ROS production and mitochondrial biogenesis. Thus, SIRT3 integrates cellular energy metabolism and ROS generation. The elucidation of the molecular mechanisms of SIRT3 regulation and its physiological functions may provide a novel target for treating ROS-related disease.

## Introduction

Peroxisome proliferator-activated receptor γ (PPARγ) coactivator-1α (PGC-1α) was originally identified as a cold-inducible transcriptional coactivator of PPARγ. Recent studies show that PGC-1α plays important roles in adaptive thermogenesis in skeletal muscle and brown fat, gluconeogenesis in liver, mitochondrial biogenesis and respiration in muscle cells, and heart development [1], [2]. Notably, PGC-1α potently induces the expression of genes implicated in energy homeostasis in almost every cell type through interacting directly with and coactivating known mitochondrial regulators, such as the estrogen related receptors (ERRs), PPARs, or nuclear respiratory factors (NRFs) [1], [2], [3], [4]. Overexpression of PGC-1α in skeletal muscle cells results in increased energy expenditure and mitochondrial biogenesis [5], [6]. PGC-1α is induced in mouse liver by fasting and regulates the expression of several key genes involved in gluconeogenesis, including phosphoenolpyruvate carboxykinase and glucose-6-phosphatase [7], [8]. PGC-1α knockout mice exhibit multiple metabolic defects, such as obesity, neurodegeneration and cardiomyopathy [9], [10].

Reactive oxygen species (ROS) can react with DNA, proteins and lipids and play important roles in many physiological and pathophysiological conditions, such as diabetes, neurodegenerative diseases, cancer, and aging [11]. Although ROS are produced in multiple cell compartments, the majority of cellular ROS (approximately 90%) contribute to mitochondrial energy metabolism. The mitochondrial electron transport chain complex I and complex III are presumed to be major sites of ROS generation [11], where electrons escape the electron transport chain and react with molecular oxygen, leading to the generation of superoxide. There are two main ways in which ROS production is limited in vivo. The first is through the action of detoxifying enzymes, including glutathione peroxidase-1 (GPx1) and superoxide dismutases (SODs). The second is through the uncoupling proteins [12], [13]. Although PGC-1α stimulates mitochondrial biogenesis and electron transport activity, it suppresses ROS production and protects neural cells from oxidative stressor-induced death through the induction of several key ROS-detoxifying enzymes. PGC-1α null mice are more sensitive to the neurodegenerative effects of oxidative stress [14].

ERRs are a family of orphan nuclear hormone receptors consisting of ERRα, ERRβ, and ERRγ [15]. The ERRs share high sequence similarity to the estrogen receptor [16]. ERRα is expressed in various tissues and participates in metabolic regulation. ERRα null mice are resistant to obesity induced by a high-fat diet and exhibit reduced lipogenesis in their adipose tissue [17]. ERRα not only is a downstream target of PGC-1α but also is coactivated by this transcriptional coactivator. Consistent with the ability of PGC-1α to induce ERRα activity and expression, these proteins both control broad aspects of mitochondrial biology, including mitochondrial biogenesis, fatty acid oxidation, and oxidative respiration [18], [19], [20].

Sirt3 is a member of the class III histone deacetylases, also referred to as sirtuins (SIRTs). The SIRTs are a conserved family of proteins possessing NAD^+^-dependent deacetylase activity, distinct from class I and II histone deacetylases. SIRTs also possess ADP-ribosyltransferase activity. SIRTs have been demonstrated to play important roles in many physiological and pathophysiological conditions, including metabolism, cell survival, cancer, aging and calorie restriction-mediated longevity of organisms ranging from yeasts to humans [21], [22]. Acetyl-CoA synthetase 2 (AceCS2), a mitochondrial enzyme that converts acetate into acetyl-CoA, was the first mitochondrial substrate of SIRT3 identified [23], [24]. Deacetylation of AceCS2 at lysine 642 by SIRT3 activates acetyl-CoA synthetase activity, providing increased acetyl-CoA to feed into the tricarboxylic acid cycle. Glutamate dehydrogenase (GDH) and a component of complex I named NDUFA9, both mitochondrial proteins involved in energy production, are deacetylated by SIRT3 [25], [26]. Ku70 and forkhead transcription factor Foxo3a are also substrates of SIRT3 [27], [28], [29]. Sirt3-deficient animals exhibit striking mitochondrial protein hyperacetylation, suggesting that SIRT3 is a major mitochondrial deacetylase [25]. Remarkably, the basal levels of ATP in the heart, kidney and liver of SIRT3 null mice are reduced more than 50%, suggesting SIRT3 plays a role in regulating energy homeostasis in vivo [26]. In addition, variability in the human SIRT3 (hSIRT3) gene was reported to be linked to human longevity [30], [31].

Human SIRT3 is a mitochondrial protein, and its N-terminal 25 amino acid residues are responsible for its mitochondrial localization [32], [33]. Human SIRT3 is synthesized as an inactive protein and is activated by matrix peptidase [33]. However, mouse Sirt3 (mSirt3) was initially reported to encode a 257 amino acid protein (approximately 28 kDa) that aligned well with the C-terminal portion of hSIRT3 (residues 143–399) [34]. Although the originally predicted mSIRT3 lacks the N-terminal 142 amino acids responsible for the mitochondrial localization of the human counterpart, it has still been shown to be localized in mitochondria [35]. In two recent studies, however, two longer forms of mouse SIRT3 (approximately 37 kDa) owning the mitochondrial target signal, were detected [36], [37].

Mouse SIRT3 is highly expressed in various tissues with high energy demands, including brain, kidney, heart and liver. Mouse SIRT3 expression is up-regulated by caloric restriction and cold exposure, and overexpression of mSIRT3 in adipocytes leads to the induction of genes involved in mitochondrial function and thermogenesis [35]. However, little else is currently known about the molecular mechanism of the regulation of Sirt3 gene expression.

Here we show that PGC-1α powerfully stimulates mSIRT3 gene expression, which is mediated by an ERR binding element mapped to the Sirt3 promoter region. Furthermore, we demonstrate that SIRT3 is required for the effects of PGC-1α on mitochondrial-related gene expression, ROS production and mitochondrial biogenesis in muscle cells.

## Materials and Methods

### Plasmids and Adenoviruses

The mSirt3 promoter (−2036 to +146) was amplified as described previously [34] using mouse genomic DNA and cloned at the *Acc65* I and *Xho* I sites of the pGL3-Basic reporter gene vector (Promega), referred to as Luc-2036. Various fragments of the 5′ flanking promoter region of mSIRT3 were generated by PCR amplification of Luc-2036 followed by cloning into pGL3-Basic using the *Acc65* I and *Xho* I sites. To amplify different promoter regions, the corresponding forward primers 5′-ATTCGGGGTACCATCCAATCCCGGTCTGGTCTAC-3′ (Luc-2036), 5′- ACATTGGTACCAGCGTCCCACTAGCCTCACGGGT-3′ (Luc-491), 5′- ATTCAGGGTACCCAAGGAGGTCGAGAGCGGCGT-3′ (Luc-242), and 5′- ATTCAGGGTACCGCTGCCAGCACCAGGCA-3′ (Luc-161) were used with the identical reverse primer 5′- ATTCAGCTCGAGCTATCTGCGAGATCCCGTGTCT-3′ (+146 bp). Site-directed mutagenesis was performed using the QuickChange kit (Stratagene, La Jolla, CA) according to the manufacturer's protocol. pcDNA3.1-PGC-1α for the expression of human full-length PGC-1α was a gift from Daniel P. Kelly (Washington University School of Medicine, St. Louis, Missouri, USA). Adenoviral vectors expressing GFP, PGC-1α, or ERRα were generated using the AdEasy system as previously described [38]. The adenoviral vector expressing short-hairpin RNAs (shRNAs) against ERRα (siERRα) was a kind gift from Dr. Anastasia Kralli (Department of Cell Biology, The Scripps Research Institute, La Jolla, California), which targets the sequence 5′-GAGCATCCCAGGCTTCTCC-3′ of mouse ERRα [39]. The sequence of siRNA against luciferase (siControl) was 5′-CTTACGCTGAGTACTTCGA-3′; the sequence of siRNA against mSirt3 (siSIRT3) was 5′- GCGTTGTGAAACCCGACAT -3′; and the sequence of siRNA against mouse PGC-1α (siPGC-1α) was 5′- GGTGGATTGAAGTGGTGTAGA-3′ [40]. Inserts were cloned into the pAdTrack-U6 shuttle vector using *Bgl* II and *Hind* III, and adenovirus constructs were created by recombination of the shuttle vector and pAdEasy vector by electroporation into BJ5183 bacteria [41]. All plasmid constructs were verified by sequencing.

### Cell Culture and Adenoviral Infection

Human embryonic kidney (HEK) 293A, HepG2, and C_2_C_12_ cell lines from ATCC were maintained in DMEM supplemented with 10% fetal bovine serum (FBS) and antibiotics. Myotube differentiation was induced as previously reported [42]. Primary mouse hepatocytes were obtained from livers of male C57BL/6 mice by collagenase perfusion as previously described [43]. All animal experiments were carried out under protocols approved by the Committee on Animal Research at the Chinese Academy of Medical Science in Beijing and were in accordance with Peking Union Medical College guidelines. Mouse hepatocytes were cultured in RPMI-1640 containing 10% FBS. Mouse hepatocytes and C_2_C_12_ myotubes were infected with adenoviruses of interest. Cells were harvested 48 h after infection to perform the various measurements.

### Luciferase Assays

HepG2 cells were cultured in 24-well plates and cotransfected with PGC-1α plasmid (0.2 µg/well), ERRα plasmid (0.2 µg/well), luciferase reporter construct (0.2 µg/well), and pRL-TK reporter plasmid (control reporter) (0.006 µg/well) according to the manufacturer's protocol (Vigorous, Beijing, China), or cells were infected with adenovirus expressing control shRNA or shRNA against ERRα. The mass of transfected plasmids was balanced with empty vector (pcDNA3.1). After transfection for 48 h, cells were harvested and measured using the Dual-Luciferase Reporter assay system (Promega), and luciferase activity was divided by the *Renilla* luciferase activity (control reporter) to normalize for transfection efficiency. All luciferase assay experiments were performed 3 times at least, and each conducted in triplicate.

### Electrophoretic Mobility Shift Assays (EMSA)

EMSA was carried out using the LightShift Chemiluminescent EMSA Kit (Pierce) as previously described [44]. Briefly, 293A cells were cultured in 100 mm dishes and then transfected with ERRα expressing plasmid (10 µg). After 24 h, the nuclear proteins were extracted according to the manufacturer's protocol (Pierce). The sequence of the wild-type oligonucleotide SIRT3-ERRE-WT was 5′- Biotin-GGTTTCTGGCCCTGCCCTTGAGGCATTA AAGAG-3′, whereas the sequence of the mutant oligonucleotide SIRT3-ERRE-mut was 5′- GGTTTCTGGCCCTGtaaTTGAGGCATTAAAGAG-3′. Equal amounts of complementary oligonucleotides were annealed. Biotin-labeled probes and protein were combined in a total volume of 20 µl reaction buffer. In competition analysis, a 200-fold excess of unlabeled competitor DNA was added to the reaction mixture. After pre-incubation for 10 min at room temperature, 20 fmol of biotin end-labeled probe was added, and the incubation was continued for an additional 20 min. The protein-DNA complexes were then resolved in a nondenaturing 6% acrylamide gel in 0.5×TBE buffer at 100 V. After electrophoresis, the gel and nylon membrane were sandwiched in a clean electrophoretic transfer unit according the manufacturer's instructions at 380 mA for 60 min. When the transfer was completed, transferred DNA was immediately cross-linked to the membrane at 120 mJ/cm^2^ for 60 seconds. Afterward, biotin-labeled DNA was detected by chemiluminescence reagent.

### Chromatin Immunoprecipitation Assays

Mouse hepatocytes were extracted or infected with adenoviruses expressing FLAG-tagged ERRα, PGC-1α or GFP as a control. After 48 h, cells were harvested in PBS and cross-linked with 1% formaldehyde, lysed, and subjected to chromatin shearing by sonication. Immunoprecipitation was carried out with anti-PGC-1α (Calbiochem) or M2 anti-FLAG antibody or normal mouse IgG (Sigma), followed by incubation with protein G-Sepharose. After chromatin immunoprecipitation, DNA was purified by phenol/chloroform extraction. PCR analysis was performed with input (2% of total immunoprecipitation) and immunoprecipitated DNA. The primers were designed to amplify the region −491 to −319 bp of the mouse SIRT3 promoter containing an ERR binding site: 5′-AGCGTCCCACTAGCCTCACGGGTTG-3′ (forward) and 5′-GAGGACCCAAGTCTGCAGGCTTGAG-3′ (reverse). The primers for the distal region (from −1400 to −1271 bp) of the mouse Sirt3 promoter were 5′-GAGACAGCGTCAACTCCCACTC-3′ (forward) and 5′-CCAATGCCTTCAAGGCTGAAG-3′ (reverse).

### RNA isolation and Quantitative Real-Time PCR

Total RNA from cultured cells was extracted using TRIzol reagent (Invitrogen). cDNA was then reverse-transcribed and amplified by PCR using a Transcriptor Reverse Transcriptase kit (Roche). Quantitative RT-PCR was carried out using the SYBR Green PCR system on a Bio-Rad iQ5, and results were normalized to β-actin or cyclophilin A. The sequences of primers are shown in table 1.

**Table 1:** Primer details.

primer	forward (5′-3′)	reverse (5′-3′)
Mouse sirt3	GCTGCTTCTGCGGCTCTATAC	GAAGGACCTTCGACAGACCGT
Human sirt3	CGGCTCTACACGCAGAACATC	CAGCGGCTCCCCAAAGAACAC
ATP5c	TCAAGTCTGTTATCTCCTAC	GAGGTTGGCCAGATTGTAC
Cyt *c*	TCTCCCCAGGTGATGCCTTT	CCAGCCTTCCTTCTTGGGTAT
SOD2	GTGAACAATCTCAACGCCA	GATAGCCTCCAGCAACTCT
GPx1	CTCGGTTTCCCGTGCAATC	CTCACCATTCACTTCGCA
Mouse β-actin	CCAGCCTTCCTTCTTGGGTAT	TGCTGGAAGGTGGACAGTGAG
Human β-actin	AGCGGGAAATCGTGCGTGAC	CGGACTCGTCATACTCCTGCT
COX I	GGATTTGTTCACTGATTCCCATTA	GCATCTGGGTAGTCTGAGTAGCG
COX II	CAGGCCGACTAAATCAAGCAAC	CTAGGACAAT GGGCATAAAG CT
COX VIIa	ATGAGGGCCCTACGGGTCTC	CATTGTCGGCCTGGAAGAG
cyclophilinA	TTCCTCCTTTCACAGAATTATTCCA	CCGCCAGTGCCATTATGG

### Western blotting

C_2_C_12_ myotubes were cultured in 60 mm dishes, infected with adenoviruses and, 48 h later, lysed in 100 µl of lysis buffer (50 mM Tris, pH 7.4, 1 mM EDTA, 150 mM NaCl, 0.25% sodium deoxycholate, and 1% NP40) containing protease inhibitors (1 mM PMSF, 1 µg/ml aprotinin, 1 µg/ml leupeptin and 1 µg/ml pepstatin) (Roche) and phosphatase inhibitors (1 mM NaF and 1 mM NaVO_3_). Samples were incubated at 4°C for 1 h and then centrifuged at 12,000 *g* for 30 min, and the supernatant was collected for analysis. Lysates were resolved by 10% SDS-PAGE and then transferred to PVDF membranes. The membranes were blocked and incubated with specific antibodies against SIRT3 (Millipore), PGC-1α (Calbiochem), SOD2 (Millipore) and actin (Sigma). Peroxidase-conjugated anti-mouse or anti-rabbit immunoglobulin secondary antibody was used. Proteins were visualized by enhanced chemiluminescence.

### Measurement of intracellular superoxide levels

Superoxide production was detected using the fluorescent dye dihydroethidium (DHE) obtained from Vigorous according to a previous study [45]. C_2_C_12_ myotubes were cultured in 6-well plates, infected with indicated adenoviruses, and 48 h later washed with PBS and labeled with DHE (5 µmol/L dissolved in 1% DMSO) in the culture plates at 37°C for 30 minutes in PBS. Culture plates were placed on ice to stop the labeling, trypsinized, and resuspended in ice-cold PBS. Samples were analyzed using a flow cytometer (COULTER EPICS®) and Expo32ADC software. The mean fluorescence intensity (MFI) of 10,000 cells was analyzed in each sample and corrected for autofluorescence from unlabeled cells.

### Mitochondrial DNA copy number

Mitochondrial DNA (mtDNA) copy number was determined as a marker for mitochondrial density using quantitative RT-PCR as previously reported [46], [47]. Briefly, total DNA was isolated from the cells using Universal Genomic DNA Extraction kit (Takara) according to the manufacturer's instructions. A serial dilution standard curve was prepared from a pool of all the samples and real-time PCR was carried out. The mitochondrial DNA copy number was calculated from the ratio of COX II (mitochondrial-encoded gene)/cyclophilin A (nuclear-encoded gene). The primers are shown in Table 1.

### Statistical Analysis

The data are reported as the mean ± S.D. of at least three independent experiments. Differences between groups were compared by Student's *t* test and *P* values<0.05 were considered statistically significant.

## Results

### PGC-1α induces mouse Sirt3 gene expression

Previous studies have suggested SIRT1 directly interacts with and deacetylates transcriptional coactivator PGC-1α [42], [48]. Additionally, overexpression of SIRT3 leads to increased PGC-1α gene expression in HIB1B brown adipocytes [35]. Both PGC-1α and sirtuins have been demonstrated to play key roles in energy metabolism. Therefore, we were prompted to study the association of PGC-1α and sirtuin family members to determine if PGC-1α affects the expression levels of sirtuin members.

**Figure 1:**
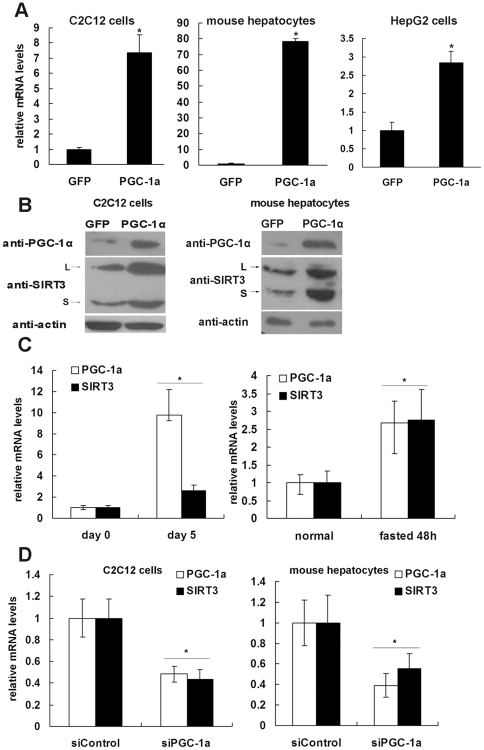
PGC-1α regulates the expression of SIRT3. **A**, Differentiated C_2_C_12_ cells (left panel), primary hepatocytes isolated from mouse liver (middle panel) and HepG2 cells (right panel) were infected with adenoviruses expressing GFP (control) or PGC-1α. Cells were harvested 48 h after infection, and total RNA was extracted. The mRNA levels of Sirt3 were quantified by qPCR, normalized to β-actin, and expressed relative to the GFP-expressing control cells. **B**, C_2_C_12_ myotubes and primary hepatocytes were treated as described in Panel A, and SIRT3 protein level was determined by western blotting. Two specific bands of SIRT3 in C_2_C_12_ (left panel) and primary hepatocytes (right panel) were detected. L, long form of mSIRT3 (approximately 37 kDa). S, short form of mSIRT3 (approximately 28 kDa). **C, left panel**, C_2_C_12_ myoblasts were induced to differentiate into myotubes. Cells were harvested on day 0 (before differentiation) and day 5 (after differentiation), and total RNA was isolated. **C, right panel**, Total RNA was extracted from normal or fasted mouse livers. The mRNA levels of PGC-1α and Sirt3 were quantified by qPCR, normalized to β-actin and expressed relative to levels in the control. **D**, Differentiated C_2_C_12_ (left panel) and primary hepatocytes (right panel) were infected with adenovirus expressing luciferase shRNA (siControl) or PGC-1α shRNA (siPGC-1α). Cells were harvested 48 hours after infection, and total RNA was extracted. The mRNA levels of Sirt3 and PGC-1α were quantified by qPCR, normalized to β-actin, and expressed relative to the control cells. *, *P*<0.05.

Differentiated C_2_C_12_ and mouse primary hepatocytes were infected with adenovirus expressing PGC-1α or GFP (control). Total RNA was extracted from infected cells, and mRNAs of sirtuin family members were measured by real-time PCR. We observed that Sirt3 mRNA levels were increased by approximately 7-fold by PGC-1α in C_2_C_12_ myotubes compared to control cells (Fig. 1A). In addition, PGC-1α stimulated Sirt3 mRNA level by approximately 70-fold and 2.8-fold in mouse primary hepatocytes and HepG2 cells, respectively (Fig. 1A). These increases in Sirt3 mRNA were accompanied by potent up-regulation of the protein levels of mSIRT3 variants in C_2_C_12_ cells and mouse primary hepatocytes (Fig. 1B). In contrast to Sirt3, PGC-1α did not alter the expression of other sirtuin members (data not shown).

PGC-1α is induced in mouse liver upon fasting [7], [8] and in C_2_C_12_ cells during differentiation [46]. Thus, we speculated that Sirt3 may be similarly regulated under these conditions. As expected, the Sirt3 mRNA level was significantly increased in myotubes compared to that in myoblasts (Fig. 1C), and fasting induced Sirt3 gene expression in mouse liver (Fig. 1C). Meanwhile, we also observed that PGC-1α was induced under these conditions, which was consistent with previous reports.

To determine the role of PGC-1α in the regulation of Sirt3 gene expression during these physiological processes, we employed a siRNA strategy to specifically knock down PGC-1α gene expression. A siRNA sequence (siPGC-1α) has been shown to effectively silence PGC-1α gene expression [40]. Adenoviruses expressing shRNA against PGC-1α were constructed and used to infect C_2_C_12_ myotubes and primary hepatocytes. Knockdown of PGC-1α in C_2_C_12_ myotubes and primary hepatocytes by siPGC-1α reduced the expression of Sirt3 (Fig. 1D). Taken together, these data indicate that PGC-1α functions as an upstream activator of Sirt3 gene expression and is required for the induction of Sirt3 in C_2_C_12_ cells and hepatocytes.

### The stimulatory effect of PGC-1α on the Sirt3 promoter is mediated by the ERR binding element located between −407 bp and −399 bp upstream of the Sirt3 transcription start site

**Figure 2:**
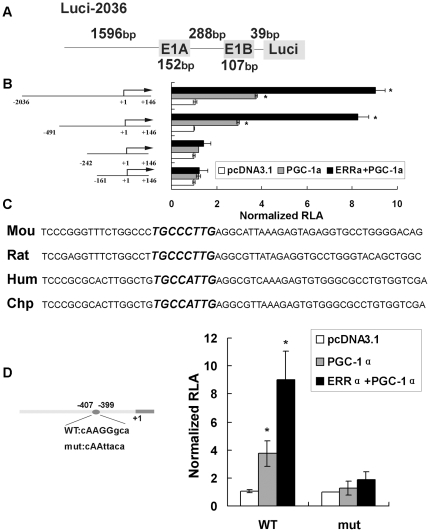
ERRα mediates the PGC-1α activation of the Sirt3 gene promoter in HepG2 cells. **A**, Schematic representation of the promoter sequence of mSirt3 (Luc-2036). Exon 1A (E1A) and exon 1B (E1B) of mSirt3 are alternatively spliced according to previous report [34]. Thus, mSirt3 has two different transcriptional start sites. However, E1A does not encode amino acids. **B**, 5′-Deletion series of the mSirt3 promoter fused to luciferase reporter gene were cotransfected into HepG2 cells together with pcDNA3.1 (control) or PGC-1α in the presence or absence of ERRα expression plasmids. Two days later, cells were harvested and the relative luciferase activity (RLA) was corrected for *Renilla* luciferase activity and normalized to the control activity. **C**, The nucleotide sequence from −424 to −366 of the mouse (Mou) Sirt3 gene promoter was aligned with corresponding sequences from different species, including rat, humans (Hum), and chimpanzee (Chp). Evolutionarily conserved elements are indicated in large, bold, italic type. **D, left panel**, Wild-type putative ERRα binding element and its mutant sequence; **right panel**, Reporter gene plasmid containing 2.2 kb of the wild-type (WT) Sirt3 promoter (Luc-2036) or ERRE mutant (mut Luc-2036) were transfected into HepG2 cells together with PGC-1α in the presence or absence of ERRα expression plasmids or empty plasmid (pcDNA3.1). The graph depicts RLA corrected for *Renilla* luciferase activity and normalized to the control activity of mSirt3. All values represent at least three independent transfections, each conducted in triplicate. *, *P*<0.05.

To demonstrate that PGC-1α-dependent stimulation of Sirt3 gene expression occurs at the transcriptional level, we transfected a promoter reporter into HepG2 cells. The first construct contained the approximately 2.2-kb fragment (−2036, +146) of the mSirt3 promoter fused to a luciferase reporter gene (Luc-2036) (Fig. 2A). Overexpression of PGC-1α caused a 4-fold activation of Luc-2036 (Fig. 2B). Next, a series of truncated segments of the promoter fused to the luciferase gene (Luc-491, Luc-242, and Luc-161) were transfected into HepG2 cells to map the cis-acting element conferring the PGC-1α-dependent activation of luciferase. PGC-1α-mediated reporter activation was not significantly compromised upon deletion of the −2036 bp to −491 bp region of the mSirt3 promoter. However, further deletion of −491 bp to −242 bp abolished the PGC-1α effect on the mSIRT3 promoter (Fig. 2B). Further truncation of the promoter from −242 bp to −161 bp did not accentuate the process (Fig. 2B).

Next, we searched for the sequence motif in the mSirt3 gene promoter that conferred sensitivity to PGC-1α. The sequence between −491 bp and −242 bp was analyzed, and a putative ERRE (CAAGGGCA) was revealed at position −407/−399 bp upstream of the transcriptional start site (Fig. 2D, left panel). In addition, we compared this motif and its flanking sequence of the mSirt3 promoter with corresponding promoter sequences from different species, including rat, humans and chimpanzee. Based on the results from sequence alignment, the putative ERR binding site located at −407/−399 bp is evolutionarily conserved (Fig. 2C). PGC-1α transactivates transcription of genes involved in oxidative phosphorylation through coactivating ERRα [49]. Thus, we hypothesized that ERRα mediates the PGC-1α stimulatory effect on the Sirt3 promoter.

Transfection of the PGC-1α expression plasmid alone in HepG2 cells activated Luci-2036 and Luci-491 each approximately 4-fold, and cotransfection of ERRα with PGC-1α activates Luci-2036 and Luci-491 each approximately 9-fold, while this effect was diminished upon further deletion of the promoter region from −491 bp to −242 bp (Luc-242) (Fig. 2B).

Mutational studies were further performed to investigate the involvement of ERR in the PGC-1α activation of the mSirt3 promoter. The potential ERR binding element was mutated from the Luc-2036 sequence (Fig. 2D, left panel), which has been demonstrated to inactivate this element previously [50]. Wild-type Luc-2036 and its mutant were transfected into HepG2 cells in the presence or absence of PGC-1α and/or ERRα. We observed that PGC-1α activated wild-type mSirt3 promoter activity and the cotransfection of PGC-1α and ERRα enhanced this effect (Fig. 2D, right panel). However, the transfection with ERRα expression plasmid alone yielded no significant increase in promoter activity (data not shown), which indicating PGC-1α and ERRα have a synergic effect. In sharp contrast, PGC-1α activation of the mSirt3 promoter was almost completely abolished upon ERRE mutation (Fig. 2D, right panel). These results clearly suggest that this ERRE mediates PGC-1α activation of mSirt3 gene expression.

### ERRα binds to the mSirt3 promoter and is required for PGC-1α- induced Sirt3 expression

**Figure 3:**
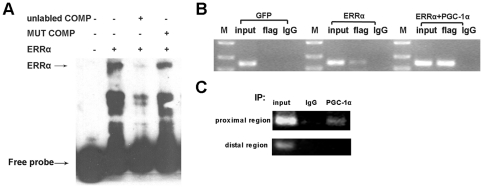
ERRα interacts with the mouse Sirt3 promoter *in vitro* and *in vivo*. **A**, Electrophoretic mobility shift assay was executed using a biotin probe. Biotin-labeled double-stranded oligonucleotides were incubated with or without nuclear extract containing ERRα protein. Approximately 200-fold excess wild-type and ERRE-mutated unlabeled double-stranded oligonucleotides were used for competitive inhibition. **B**, Primary hepatocytes were isolated, cultured in 100 mm dishes, and infected with adenoviruses expressing ERRα-FLAG and/or PGC-1α, or GFP as a control. For ChIP, protein-DNA complexes were immunoprecipitated with anti-FLAG or control IgG antibody. The mSirt3 promoter region harboring the ERRE site was amplified by PCR. **C**, Primary hepatocytes were isolated from mouse liver, and chromatin was immunoprecipitated with anti-PGC-1α antibody. Normal IgG was used as control. The mSirt3 promoter region harboring the ERRE (proximal region) could be amplified by PCR. However, the distal region of the mSirt3 promoter, having no ERRE and used as a negative control, could not be amplified.

To determine whether ERRα binds to the potential binding element identified in the mSirt3 gene promoter *in vitro*, a DNA fragment encompassing the sequence −386/−419 was biotin-labeled, and EMSA was carried out using nuclear protein extracted from 293A cells transfected with ERRα expression vector. A retardation band (DNA-protein complex) was specifically detected, and it almost completely disappeared upon the use of 200-fold excess of unlabeled wild-type oligonucleotides. However, 200-fold excess of unlabeled mutant oligonucleotides did not affect the formation of the DNA-protein complex (Fig. 3A).

To further confirm that ERRα binds to the Sirt3 promoter *in vivo*, chromatin immunoprecipitation assays were performed. To this end, mouse primary hepatocytes were isolated and infected with adenoviruses expressing GFP or ERRα-FLAG in the absence or presence of PGC-1α. Chromatin was precipitated with anti-FLAG or normal mouse IgG (negative control). Primers were designed to amplify a fragment of the ERRE flanking region. A fragment of the promoter (−319/−491) was PCR amplified. As shown in Figure 3B, the mSirt3 promoter fragment containing the ERRE was amplified from the precipitates using FLAG-specific antibody but not from those with normal mouse IgG in cells infected with Ad-ERRα-FLAG. Moreover, no amplification was observed in cells infected with Ad-GFP. It is noteworthy that chromatin isolated from cells co-infected with Ad-PGC-1α and Ad-ERRα-FLAG resulted in much stronger amplification of the Sirt3 promoter containing the ERRE than that from cells infected with Ad-ERRα-FLAG alone, indicating PGC-1α increased ERRα binding to the Sirt3 promoter *in vivo*.

To determine whether endogenous PGC-1α occupies the mSirt3 promoter region in primary hepatocytes and whether this is dependent on the ERRE in the gene promoter, ChIP was performed and chromatin was precipitated with anti-PGC-1α antibody. The mSIRT3 promoter sequence containing the ERRE (proximal region) could be amplified from the precipitates obtained using the anti-PGC-1α antibody, but not from the control IgG sample (Fig. 3C). As expected, the distal promoter region without an ERRE, used as a negative control, could not be amplified from any of the immunoprecipitates. Collectively, these data confirm the interaction of ERRα with an ERRE identified in the mSirt3 promoter *in vivo* and *in vitro*, and they reveal that endogenous PGC-1α can be recruited to the proximal region containing this ERRE.

**Figure 4:**
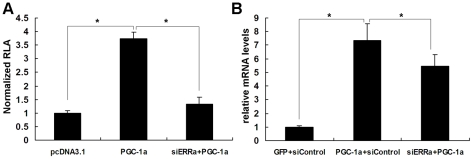
ERRα is required for the PGC-1α induction of Sirt3 expression. **A**, The full-length wild-type Sirt3 promoter fused to luciferase reporter gene (Luc-2036) was cotransfected into HepG2 cells with pcDNA3.1 (control) or PGC-1α in the presence of shRNA against ERRα or luciferase (control). Two days later, cells were harvested and RLA analyzed. **B**, Knockdown of ERRα reduced the induction of Sirt3 mRNA level by PGC-1α. C_2_C_12_ myotubes were infected with adenoviruses expressing GFP or PGC-1α in the presence of Ad-siControl or Ad-siERRα. Total RNA was isolated 48 h later. Sirt3 mRNA level was determined by qPCR, normalized to β-actin, and expressed relative to control cells infected with Ad-GFP and Ad-siControl. All values represent at least three independent transfections, each conducted in triplicate. *, *P*<0.05.

To further determine the role of ERRα in the PGC-1α induction of Sirt3 expression, we used siRNA oligonucleotides that have been demonstrated to specifically knock down ERRα gene expression [39]. As shown in Fig. 4A, ERRα knockdown compromised the ability of PGC-1α to activate the mSirt3 promoter. Moreover, siRNA against ERRα reduced the PGC-1α induction of endogenous mSirt3 mRNA level in C_2_C_12_ cells (Fig. 4B). We observed that PGC-1α still stimulated Sirt3 gene expression after knocking down ERRα, which may have resulted from decreased but not completely abolished ERRα expression by the siRNA. These results clearly indicate that ERRα plays an important role in mediating the PGC-1α induction of Sirt3 expression.

### Sirt3 knockdown suppresses PGC-1α-induced mitochondrial-related gene expression

**Figure 5:**
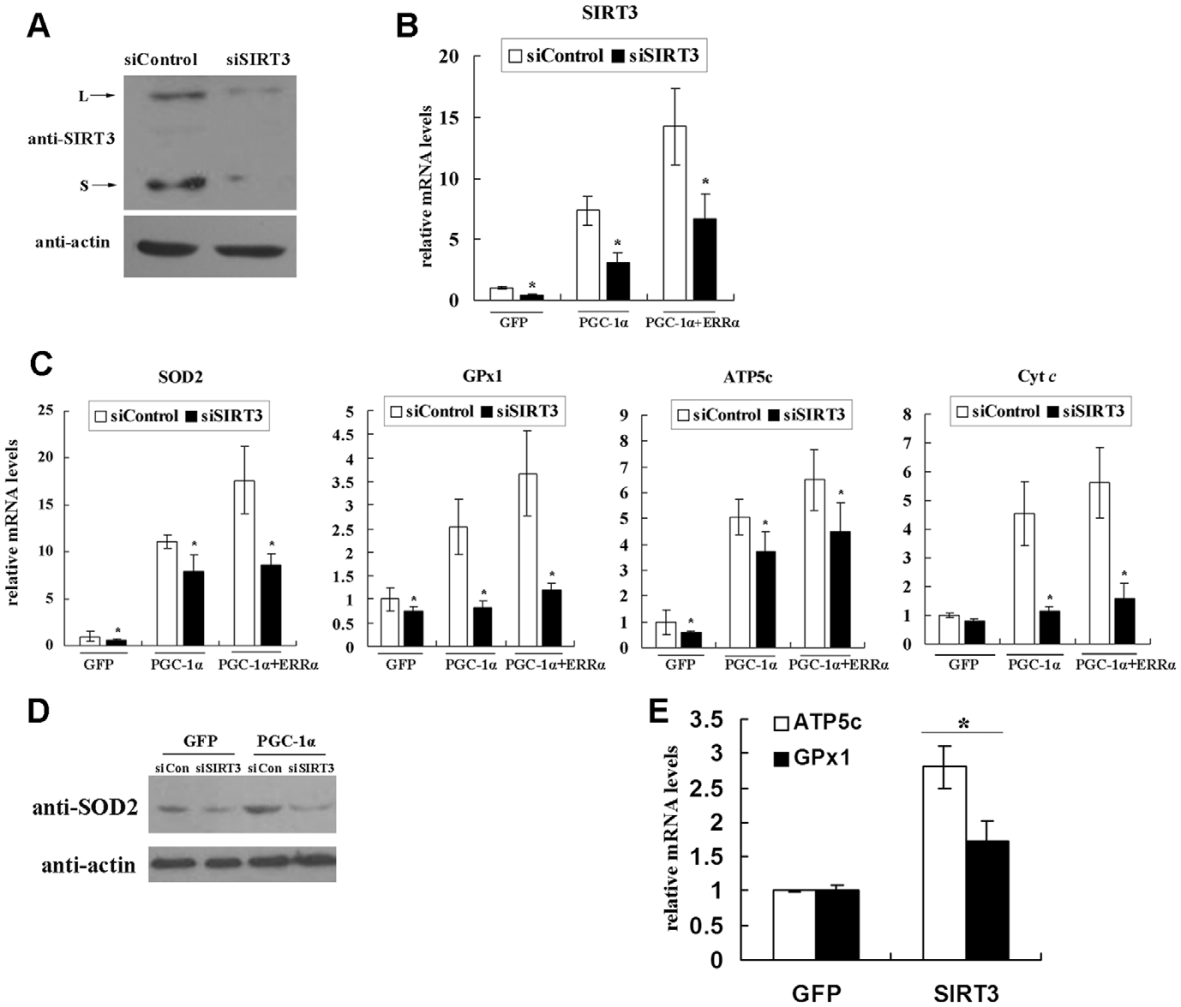
SIRT3 mediates the PGC-1α induction of mitochondrial-related genes in C_2_C_12_ skeletal muscle cells. **A**, Knockdown of mSIRT3 protein level with shRNA in C_2_C_12_ myotubes. C_2_C_12_ cells were induced to differentiate into myotubes and infected with adenovirus expressing either control shRNA (siControl) or SIRT3 shRNA (siSIRT3). Cells were harvested 48 h after infection, and protein was extracted for western blotting with the indicated antibodies. **B**, mSirt3 mRNA level was reduced by shRNA against mSirt3 in C_2_C_12_ myotubes. C_2_C_12_ myoblasts were induced into myotubes and infected with the indicated adenovirus expressing siSIRT3 or siControl, in the presence of Ad-GFP or Ad-PGC-1α and/or Ad-ERRα. Total RNA was extracted 48 h after infection, and Sirt3 mRNA level was determined by qPCR, normalized to β-actin, and expressed relative to control cells infected with Ad-GFP. **C**, Knockdown of SIRT3 decreases the PGC-1α induction of mitochondrial-related gene expression. C_2_C_12_ cells were treated as described in Panel B. The mRNA levels of SOD2, GPx1, ATP5c and Cyt *c* were measured by qPCR. **D**, C_2_C_12_ cells were treated as described in Panel B. The protein levels of SOD2 were determined by western blotting. **E**, Overexpression of SIRT3 increased the mRNA levels of GPx1 and ATP5c. C_2_C_12_ myotubes were infected with adenoviruses expressing GFP or long-form mSIRT3. Cells were harvested and total RNA was extracted 48 h after infection. The mRNA levels of GPx1 and ATP5c were measured by qPCR, normalized to β-actin, and expressed relative to control cells infected with Ad-GFP. Values represent the mean of three independent experiments performed in triplicate. *, *P*<0.05.

PGC-1α activates mitochondrial-related gene expression in skeletal muscle cells, including ATP synthase and cytochrome *c*, which are important components of the electron transport chain, as well as SOD2 and GPx1, which are involved in ROS metabolism [6]. Mouse SIRT3 is localized in the mitochondria and associated with energy metabolism [26], [35]. We wondered whether SIRT3 mediates the PGC-1α effect on the expression of these mitochondrial-related genes in myotubes. Adenoviruses expressing shRNAs targeting mSirt3 were constructed. The shRNA were expressed effectively under the control of a U6 RNA promoter. An efficient shRNA sequence (siSIRT3) for silencing mSirt3 expression was selected from several candidates. siSIRT3 decreased basal SIRT3 protein level by more than 60% compared with the luciferase shRNA (negative control) (Fig. 5A). siRNA against mSirt3 significantly suppressed the PGC-1α induction of endogenous Sirt3 mRNA level (Fig. 5B). Therefore, this effective siRNA was employed in the following experiments. C_2_C_12_ myotubes were infected with various combinations of adenoviruses. As expected, PGC-1α activated some mitochondrial-related genes, including ATP synthase, cytochrome *c*, and the ROS-detoxifying enzymes GPx1 and SOD2. Moreover, coinfection with Ad-ERRα and Ad-PGC-1α led to further enhancement of these gene expression levels (Fig. 5C). In contrast, PGC-1α-induced mitochondrial gene expression was largely prevented upon knockdown of Sirt3 in C_2_C_12_ cells (Fig. 5C). However, the control siRNA did not affect the PGC-1α activation of these genes. In addition, we measured SOD2 protein levels in C_2_C_12_ myotubes infected with various combinations of adenoviruses. As shown in Figure 5D, siSIRT3 decreased the baseline protein level of SOD2 and reduced the induction of SOD2 by PGC-1α (Fig. 5D).

Overexpression of Sirt3 in cardiomyocytes is capable of modulating the activity of antioxidants [27]. An adenovirus expressing the long version of mSirt3 was constructed and used to infect C_2_C_12_ myotubes. As anticipated, overexpression of Sirt3 in C_2_C_12_ myotubes increased the mRNA levels of ATP5c and GPx1, but not SOD2 or Cyt *c*. (Fig. 5E). These data demonstrate that Sirt3 functions as a downstream target gene of PGC-1α and mediates, at least in part, the effects of PGC-1α on the ROS defense system.

### SIRT3 mediates the effects of PGC-1α on intracellular ROS levels and mitochondrial biogenesis

SIRT3 is localized in the inner mitochondrial membrane [32] along with the electron transport chain. PGC-1α is suggested to be a potent stimulator of mitochondrial biogenesis [5], [6] and a powerful regulator of ROS metabolism. Although PGC-1α stimulates mitochondrial biogenesis and energy metabolism, it suppresses ROS production in cells through induction of ROS-detoxifying enzymes [14]. The mitochondrion is the major site of superoxide formation, and the accumulation of superoxide is believed to contribute to oxidative damage associated with degenerative disease and aging [51]. Since knockdown of Sirt3 suppressed the PGC-1α induction of ROS-detoxifying enzymes, it seemed logical to propose that SIRT3 mediates the PGC-1α-dependent down-regulation of intracellular superoxide levels.

**Figure 6:**
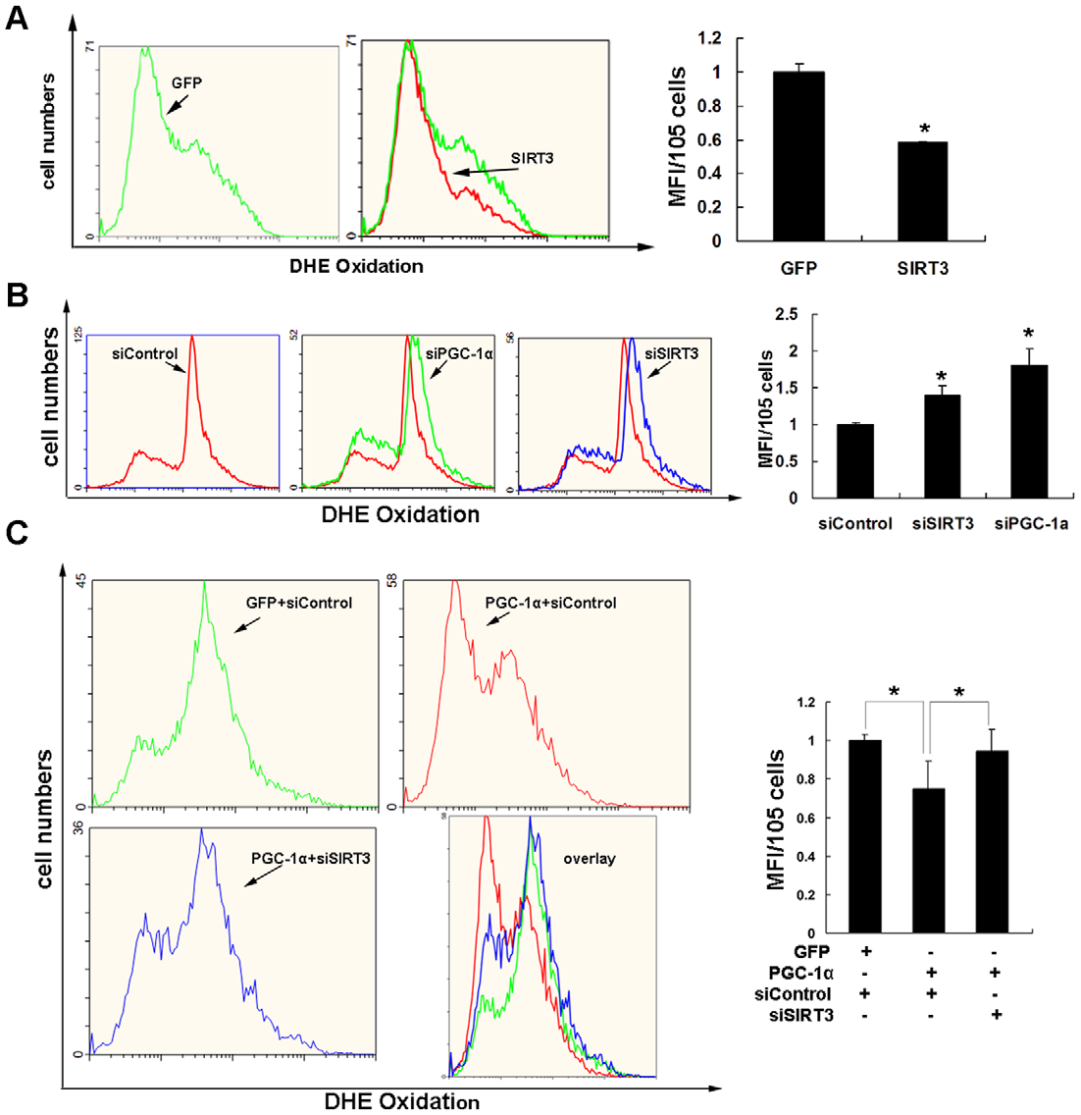
SIRT3 controls intracellular ROS levels. **A**, C_2_C_12_ myotubes were infected with adenovirus expressing GFP or SIRT3. DHE (5 µM) was added to media 30 min before collecting cells. The cells were harvested and immediately analyzed with a flow cytometry, and data were processed with Expo 32ADC software. The histogram overlays showed overexpression of mSIRT3 reduced the cellular ROS level. **B**, Knockdown of PGC-1α or SIRT3 increased cellular ROS level. C_2_C_12_ myotubes were treated with the indicated adenovirus (Ad-siControl, Ad-siPGC-1α or Ad-siSIRT3), treated with DHE, and analyzed with a flow cytometry as described in panel A. **C**, Overexpression of PGC-1α decreased cellular ROS level, and knockdown of SIRT3 blocked the inhibitory effect of PGC-1α on ROS production. C_2_C_12_ myotubes were treated with the indicated adenovirus (Ad-GFP, Ad-PGC-1α, Ad-siControl, or/and Ad-siSIRT3), treated with DHE, and analyzed with a flow cytometer as described in panel A. *x*-Axis, fluorescence intensity showing the extent of DHE oxidation; *y*-axis, cell number. The image is representative of three experiments. **Right panels**, Flow cytometry analysis of myotube cells. Values represent the mean of four independent experiments performed in duplicate. MFI, mean fluorescent intensity. *, *P*<0.05.

As shown in Figure 6A, the basal superoxide level was decreased in C_2_C_12_ myotubes infected with adenovirus expressing Sirt3 compared with control cells as evaluated with ROS-activated fluorescent dye DHE, suggesting SIRT3 plays an important role in ROS production. Meanwhile, inhibition of endogenous PGC-1α or SIRT3 by adenovirus expressing shRNA in C_2_C_12_ myotubes caused an increase of cellular ROS production (Fig. 6B). In contrast, overexpression of PGC-1α in C_2_C_12_ myotubes with adenovirus caused a decrease of ROS production (Fig. 6C). Interestingly, knockdown of Sirt3 gene expression with adenoviruses rescued the inhibitory effect of PGC-1α on ROS production, and the intracellular ROS level was almost completely recovered, which suggests SIRT3 mediates the PGC-1α suppression of ROS production in muscle cells (Fig. 6C).

**Figure 7:**
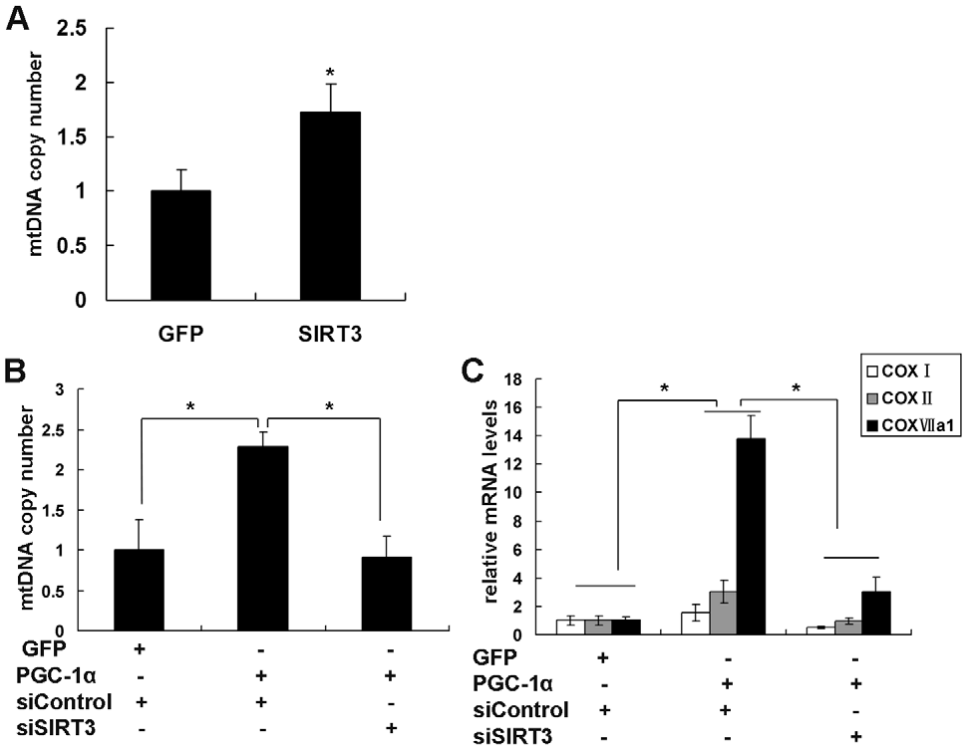
SIRT3 mediates the PGC-1α induction of mitochondrial biogenesis in C_2_C_12_ myotubes. **A**, SIRT3 overexpression stimulated mitochondrial biogenesis. C_2_C_12_ myotubes were infected with Ad-GFP or Ad-SIRT3. Cells were harvested 48 h after infection, and the DNA level of the mitochondrial-encoded COX II gene was measured by qPCR, normalized to DNA levels of the nuclear-encoded gene cyclophilin A, and expressed relative to levels in control cells expressing GFP, which were set to 1. **B**, Knockdown of SIRT3 inhibited the induction of mitochondrial biogenesis by PGC-1α. C_2_C_12_ myotubes were infected with the indicated adenovirus (Ad-GFP, Ad-PGC-1α, Ad-siControl, or/and Ad-siSIRT3). Mitochondrial DNA copy number was measured as described in panel A. **C**, Knockdown of SIRT3 inhibited the induction of mRNA level of COX I, COX II and COX VIIa by PGC-1α. C_2_C_12_ myotubes were infected with the indicated adenovirus (Ad-GFP, Ad-PGC-1α, Ad-siControl, and/or Ad-siSIRT3). Cells were harvested and total RNA was extracted 48 h after infection. The mRNA levels of mitochondrial-encoded (COX I and COX II) and nuclear-encoded (COX VIIa) subunits of COX were measured by qPCR, normalized to β-actin, and expressed relative to control cells infected with Ad-GFP and Ad-siControl. Values represent the mean of three independent experiments performed in duplicate. *, *P*<0.05.

We also evaluated the role of SIRT3 in the PGC-1α effect on mitochondrial biogenesis. To measure mitochondrial DNA directly, we isolated total DNA and determined the relative copy number of mitochondrial DNA by a qPCR assay of the mitochondrial DNA–encoded COX II gene. Adenovirus-mediated overexpression of SIRT3 led to an increase in mitochondrial DNA content per cell by 1.7-fold (Fig. 7A), while inhibition of endogenous SIRT3 by shRNA did not change the basal mitochondrial DNA content (data not shown). As anticipated, overexpression of PGC-1α also increased the mitochondrial DNA copy number (Fig. 7B). Furthermore, knockdown of endogenous mSIRT3 reduced the induction of mitochondrial biogenesis by PGC-1α (Fig. 7B). In addition, mRNA transcript levels of cytochrome *c* oxidase (COX) subunits I and II (mitochondrial-encoded) and subunit VIIa (nuclear- encoded) were induced by PGC-1α (Fig. 7C). Knockdown of endogenous mSIRT3 blocked the PGC-1α effect on COX I, COX II and COX VIIa1 gene expression (Fig. 7C). These results reveal the important physiological role of SIRT3 in mitochondrial biogenesis.

**Figure 8:**
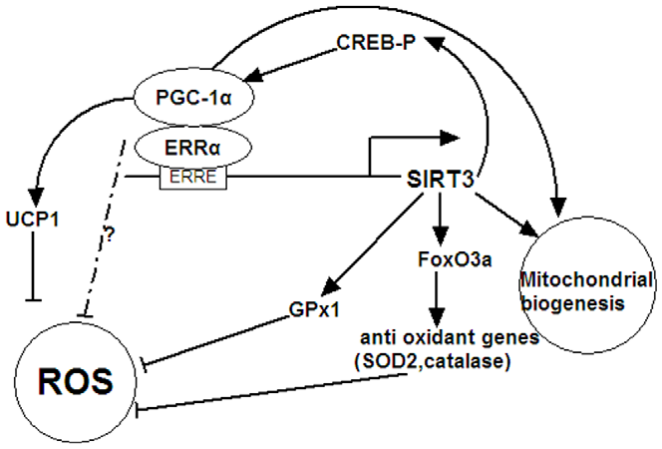
Scheme illustrating a regulatory pathway including PGC-1α and SIRT3 governing ROS level and mitochondrial biogenesis. PGC-1α coactivates ERRα to stimulate the expression of mSIRT3, which, in turn, increases the expression of the ROS-detoxifying enzymes GPx1 and SOD2 to suppress the ROS levels. Meanwhile, ectopic expression of SIRT3 leads to an increase of CREB phosphorylation, which subsequently stimulates the expression of PGC-1α and its target gene UCP1, finally decreasing intracellular ROS level. SIRT3 is also involved in mitochondrial biogenesis.

Previous work has suggested overexpression of SIRT3 in brown adipocytes leads to increased CREB phosphorylation, subsequent stimulation of the expression of PGC-1α and its target gene UCP1, and, ultimately, decreased intracellular ROS level [35]. Another study demonstrated that PGC-1α suppresses ROS production through the induction of ROS-detoxifying enzymes [14]. Finally, SIRT3 interacts with and deacetylates Foxo3a to activate its target gene SOD2 in cells [27]. Based on these data and our observations, we propose a model of the mechanism of SIRT3 action on ROS production and mitochondrial biogenesis (Fig. 8). In this model, SIRT3 stimulates PGC-1α gene expression through increased CREB phosphorylation. Meanwhile, PGC-1α induces SIRT3 expression through ERRα, thus forming a positive-feedback loop. In addition to suppression of basal ROS production in cells, our data clearly demonstrate that SIRT3 mediated the PGC-1α induction of ROS-detoxifying enzymes and reduction of ROS level. Finally, our results also reveal a physiological role for SIRT3 in mitochondrial biogenesis.

## Discussion

Sirtuins are NAD^+^-dependent enzymes that have been implicated in a wide range of physiological and pathophysiological conditions, including diabetes, cancer, life span and neurodegeneration, through deacetylation of numerous substrates [52]. Seven mammalian sirtuin members have been identified to date. One well-studied sirtuin, SIRT1, is reported to interact with and deacetylate PGC-1α to increase its activity, subsequently leading to the induction of liver gluconeogenic genes and the promotion of mitochondrial oxidative phosphorylation and fatty acid oxidation in muscle cells [42], [48].

SIRT3 is a soluble mitochondrial protein controlling global mitochondrial protein acetylation levels. Sirt3 knockout mice show striking mitochondrial protein hyperacetylation [25]. Furthermore, SIRT3 deacetylates AceCS2, GDH, Fox3a and Ku70 to regulate their activity [23], [24], [25], [27], [28], [29]. These observations implicate SIRT3 in the regulation of energy metabolism. Thus, we decided to further investigate the association between PGC-1α and sirtuin members.

Our data suggest that PGC-1α stimulates SIRT3 expression at the mRNA and protein levels. We did not observe an effect of PGC-1α on other sirtuins. PGC-1β, another member of the PGC-1 family, mildly stimulated SIRT3 gene expression (approximately 2-fold) in C_2_C_12_ cells (data not shown), much less effectively than PGC-1α. We speculate that PGC-1β also activates Sirt3 transcription through the ERRE identified in the mSirt3 gene promoter because PGC-1β interacts with and coactivates ERRα to stimulate downstream target genes [20].

Current studies of SIRT3 mainly focus on its cellular localization and identification of its substrates. However, the molecular mechanism regulating Sirt3 gene expression remains unknown. Our data demonstrate the existence of a regulatory pathway that drives mitochondrial ROS generation and mitochondrial biogenesis that is defined by PGC-1α, ERRα and SIRT3. This is supported by the following evidence: First, PGC-1α increased the expression of SIRT3 mRNA and protein. PGC-1α knockdown by shRNA reduced Sirt3 mRNA level. Second, the stimulatory effect of PGC-1α depended on the intact ERR binding site in the mSirt3 promoter, and cotransfection of PGC-1α and ERRα had a synergic effect on mSIRT3 promoter activity. Interestingly, overexpression or inhibition of ERRα had no effect on Sirt3 promoter activity or mRNA level in the absence of PGC-1α, leading us to conclude that ERRα contributes to the PGC-1α-mediated induction but not the basal expression of Sirt3. Third, EMSA and ChIP demonstrated ERRα binding to the ERRE identified in the Sirt3 promoter *in vitro* and *in vivo*, and PGC-1α further enhanced ERRα binding to the Sirt3 promoter *in vivo*. Fourth, induction of mitochondrial-related gene expression, including the ROS-detoxifying enzymes SOD2 and GPx1, by PGC-1α was impaired by Sirt3 knockdown in C_2_C_12_ myotubes. Fifth, knockdown of SIRT3 diminished the effect of PGC-1α on cellular ROS level and mitochondrial biogenesis. Finally, PGC-1α action explains the stimulatory effect of C_2_C_12_ differentiation and nutrient treatment in mice on Sirt3 expression. Taken together, our data suggest that Sirt3 is a new downstream target gene of PGC-1α and that SIRT3, at least in part, mediates the effects of PGC-1α on mitochondrial metabolism.

Mitochondria are a major source of superoxide formed by the reaction of respiratory chain enzymes with molecular oxygen. PGC-1α stimulates mitochondrial energy metabolism and mitochondrial biogenesis through coactivation of several transcription factors. PGC-1α increases the expression of most, if not all, mitochondrial proteins, which could cause an increase of ROS production. However, PGC-1α can induce the expression of genes reducing ROS formation [6], [53], [54]. St-Pierre et al. reported that PGC-1α is required for the induction of ROS-detoxifying enzymes under oxidative stress conditions and that PGC-1α–null mice are more sensitive to oxidative stress affecting the hippocampus [14]. Our data demonstrate that PGC-1α is a suppressor of ROS generation in skeletal muscle cells by increasing the expression of GPx1 and SOD2. We also show that knockdown of endogenous SIRT3 expression led to an increase of cellular ROS level and that overexpression of SIRT3 decreased the ROS level, indicating that SIRT3 acts as suppressor of ROS formation like PGC-1α *in vivo* and mediates the effect of PGC-1α on cellular ROS production. These results are consistent with a previous report that constitutive expression of SIRT3 reduces ROS production in brown adipocytes [35]. Longevity is a complex phenomenon, and the sirtuins have been shown to play a role in the genetic regulation of longevity. Of interest, it has been reported that polymorphism in the hSirt3 gene is linked to longevity [30], [31]. In tumor cells, hSIRT3 is identified as a cell survival factor that protects cells from genotoxic stress [55]. However, less is currently known about the mechanism of SIRT3-mediated cell protection during stress. Our results suggest that SIRT3 can inhibit the formation of ROS, which are implicated in aging and cancer. Thus, we speculated that the functional role of SIRT3 in longevity and cell survival contributes to its suppression of the ROS level.

PGC-1α induces mitochondrial biogenesis through coactivation of NRF-1 and NRF-2 to increase mitochondrial transcription factor A (mtTFA) [5]. We found that inhibition of SIRT3 expression by shRNA diminished the PGC-1α induction of mitochondrial biogenesis. A possible explanation is that NRF-1 and mtTFA, which localize to mitochondria, are also substrates of SIRT3 and their activities are regulated by SIRT3. Further studies are required to determine the molecular mechanism by which SIRT3 controls mitochondrial biogenesis. SIRT3-null mice exhibit mitochondrial protein hyperacetylation [25] and reduced basal levels of ATP in the heart, kidney and liver tissues [26], but these reports did not indicate whether mitochondrial DNA number is reduced in these tissues of Sirt3-knockout mice. In this study, we also provide evidence that SIRT3 is necessary for PGC-1α induction of Cyt *c* and ATP synthase, both of which are key components of the electron transport chain. It is logical to propose that SIRT3 participates in lipid acid oxidation and mitochondrial oxidative phosphorylation.

Human SIRT3 has two different forms, a full-length protein (approximately 44 kDa) and a shorter, active form (approximately 28 kDa) lacking the N-terminal 142 amino acids [33], [56]. However, the molecular weight of mouse SIRT3 is controversial. Sundaresan *et al.* reported that the full-length mSIRT3 protein (precursor) is 44 kDa [28], while Cooper et al. and Jin et al. reported that the longer variant should be 37 kDa [36], [37]. Since the long variant of mSIRT3 consists of 334 amino acids, we calculate that the longer variant should be 37 kDa. Our western blot results confirmed that there were two forms of endogenous mSIRT3 (37 kDa and 28 kDa) detected by the antibody recognizing the C-terminus of mSIRT3.

In addition, the subcellular localization of SIRT3 also remains controversial. Initial studies suggested that full-length and processed forms of hSIRT3 are localized in mitochondria [32], [33], but another study indicated that full-length hSIRT3 is localized in the nucleus and that processed forms are localized in the mitochondria [56]. Mouse SIRT3 was originally identified to be localized in the mitochondria [35]; in contrast, another recent study showed that the full-length form of mSIRT3 is present in the mitochondria and the 28-kDa form is detectable in the mitochondria, nucleus and cytoplasm [36], [37]. We detected mSIRT3 protein in both nucleus and mitochondria in C_2_C_12_ cells, and both nuclear and mitochondrial mSIRT3 were induced by PGC-1α (data not shown).

Overexpression of SIRT3 in brown adipocytes activates CREB phosphorylation, resulting in stimulation of PGC-1α and UCP1 gene expression, consequently decreasing the cellular ROS level. Another recent study reported that SIRT3 can deacetylate Foxo3a to activate its target gene SOD2 [27], [29]. In addition, PGC-1α suppressed ROS production through induction of ROS-detoxifying enzymes [14]. We summarize these results and propose a model of the mechanism of SIRT3 action on ROS production and mitochondrial biogenesis. As shown in Figure 8, PGC-1α induces SIRT3 expression through coactivation of ERRα, and in turn, SIRT3 stimulates PGC-1α gene expression through activation of CREB phosphorylation, thus forming a positive-feedback loop. In addition to suppression of basal ROS production in cells, SIRT3 mediates the induction of ROS-detoxifying enzymes and reduction of ROS level by PGC-1α. Finally, our results also reveal the physiological role of SIRT3 in mitochondrial biogenesis.

ROS-related damage has been implicated in cancer, aging, diabetes, and neurodegenerative diseases, and SIRT3 plays important roles in reducing cellular ROS level. As a regulatory enzyme, SIRT3 may be used as a therapeutic target to treat these ROS-related diseases.
